# Current Challenges of Organ Donation Programs in Syria

**Published:** 2010-02-01

**Authors:** B. Saeed

**Affiliations:** *Kidney Hospital, Kidney Transplant Department, Damascus, Syria*

**Keywords:** Kidney transplantation, Syria, end-stage renal disease, organ donation

## Abstract

Background: Renal transplantation is the optimal treatment for the majority of patients with end-stage renal disease.

Objective: To examine the donor characteristics of kidney transplants in Syria and the impact of national Syrian legislation on the evolution of kidney transplantation activities in the private and public sectors.

Methods: Available data on all kidney transplants performed in Syria over the last 2 decades was retrospectively analyzed to assess the characteristics of kidney donors and recipients with a focus upon transplants since 2003.

Results: The kidney transplant rate has increased from 7 kidney transplants per million populations in 2002 to more than 17 in 2007. In the meantime, a substantial decline in the rate of kidney transplantation performed on Syrian nationals abroad was observed from 65% of all kidney transplantations in 1998 to less than 2% in 2007. Despite the prohibition to buy a kidney in Syria, vendors had found ways to sell their kidneys through disreputable brokers. Potential related donors were not inclined to donate kidneys to their relatives as long as kidneys could be bought from a non-related donor. By 2008, the percent of related donors in private sector represented only 8% of all donors, as compared to 50% in public hospitals. Consequently, in January 2008, the government of Syria issued a pronouncement restricting kidney transplantation to the public sector with a new national regulatory oversight of transplantation practices. Since this 2008 Administrative Order was promulgated, the kidney transplant rate in public hospitals has substantially increased by 55% with the establishment of new public transplant centers in the 3 largest cities in Syria.

Conclusion: The recommendations of the Istanbul Declaration and the Revised Guiding Principles of the World Health Organization have yet to be implemented in Syria but the expansion of kidney transplants in the public sector is an important initial step for initiating a deceased organ donation program as an essential component of a comprehensive approach to the problem of the organ shortage.

## INTRODUCTION

Renal transplantation is the optimal treatment for the majority of patients with end-stage renal disease (ESRD) [1, 2]. There is no conclusive argument that could justify, from an ethical point of view, a general exclusion of unrelated donors [3-7].

Organs from unrelated donors are becoming a viable way, in many parts of the world, to lessen the organ shortage. The kidney transplant program in Syria has exclusively been relying on living related donors and remained sluggish for more than two decades. Considering the widening gap between the demand and supply of kidneys and other organs, in November 2003, a new national Syrian legislation was enacted and authorized the use of organs from both volunteer strangers and deceased donors for transplantation with a national guidance on organ transplantation which stated that the donation act must be altruistic and between nationals to avoid transplant tourism. That legislation has been considered as a landmark in the history of organ transplantation in Syria, since it recognized, for the first time, the concept of brain death and authorized retrieving organs from deceased donors. In January 2008, the government of Syria issued a pronouncement restricting kidney transplantation to the public sector with a new national regulatory oversight of transplantation practices.

The objective of this study was to examine the kidney donor characteristics in Syria, the impact of national Syrian legislation on the evolution of kidney transplantation activities in the private and public sectors, and how the recent restriction of kidney transplantation to public hospitals has changed the volume and profiling of kidney transplantation nationwide.

## METHODS

Available data on the numbers of all kidney transplantations performed for Syrian nationals in the private and public sector hospitals in Syria and abroad from 1990 to 2008 was retrospectively analyzed with a focus upon transplants beginning 2003. We reviewed the medical records of all donors from kidney transplant centers in Damascus over a period of three years (2005–2007) and recorded the donors and recipients’ genetic relationship if any, their ages, genders, and differences in age. The different barriers to donate kidney from 152 related potential donors were also recorded from the Kidney Hospital which is a public transplant center in Damascus.

## RESULTS

The kidney transplant rates in Syria over the last two decades were around two transplants per million population (pmp) in the 1990s, 7.5 pmp in 2002, and 13 pmp in 2008 (Table 1). It should be mentioned that the Syrian population is twenty millions. In the meantime, the number of kidney transplantation performed on Syrians undergoing kidney transplant abroad was 103 (65% of all kidney transplantations) in 1998, eight (<2%) in 2007, and five (<2%) in 2008 (Table 1). The total number of kidney transplants in public hospitals averaged 33 (19–52) or around 2 pmp per year in the 1990s, 127 (6.5 pmp) in 2002, 162 (8 pmp) in 2007, and 252 (12.5 pmp) in 2008. The number of kidney transplants in private hospitals was 24 (1 pmp) in 2002, and 188 (9.5 pmp) in 2007 (Table 1).

**Table 1:** Number of kidney transplants performed in Syria: in *vs* out of the country and public *vs* private hospital statistics

**Year**	**1990**	**1991**	**1992**	**1993**	**1994**	**1995**	**1996**	**1997**	**1998**	**1999**	**2000**	**2001**	**2002**	**2003**	**2004**	**2005**	**2006**	**2007**	**2008**
Number of kidney transplant in Syria	31	21	19	51	14	19	30	43	54	54	73	86	151	227	221	260	339	350	259
Number of Syrians undergoing kidney transplant abroad	16	12	19	21	40	15	20	34	103	78	57	58	37	15	9	9	5	8	5
Number of kidney transplants in the public hospitals	31	21	19	51	14	19	30	43	50	52	62	69	127	180	144	168	181	162	252
Number of kidney transplant in the private hospitals	0	0	0	0	0	0	0	0	4	2	11	17	24	47	77	92	158	188	7

**Figure 1:**
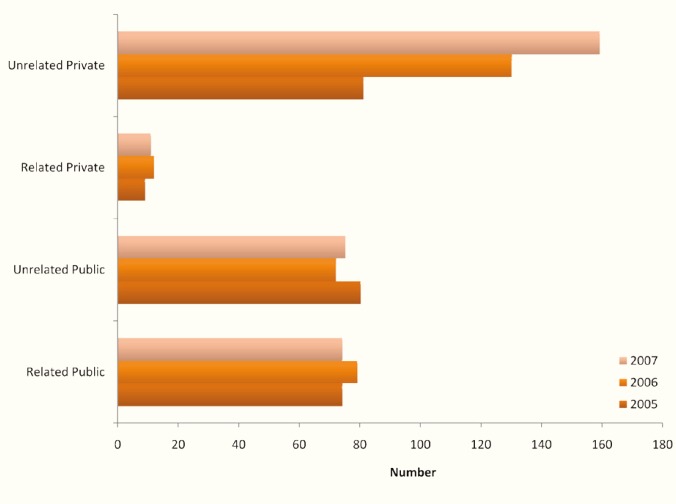
Number of related and unrelated kidney donors in Damascus public and private hospitals from 2005–2007

A total of 856 kidney donors were studied, of whom 454 (53%) had been operated in public hospitals and 402 (47%) in private hospitals. Six-hundred and eight (71%) donors were unrelated to their recipients. Further analyzing revealed that in private hospitals, unrelated donors accounted for 90%, 92%, and 96% of all donors in 2005, 2006, and 2007, respectively with an average of 92% (370 of 402 donors), whereas in public hospitals, unrelated donors accounted for 50% of all the donors (227 of 454 donors) (Fig. 1). Males accounted for 76% (n=653) of all donors and 87% (n=529) of unrelated donors. Ninety-one percent (n=553) of unrelated donors were younger than 40 years, of whom 60% (n=334) were 30–40 years old, whereas 31% (n=77) of related donors were older than 40 years. However, 45% (n=112) of related donors were 20–30 years old. Forty-six percent (n=394) of the donors were younger than their recipients with a mean difference in age of 14 years, and 54% of the donors (n=462) were older than their recipients with a mean difference in age of 12 years. Male to male donations were encountered in 323 cases (40%); female to female donations in 94 cases (11%); and both male to female and female to male donations were each seen in almost 25% of the cases (n=221 and n=218, respectively).

The barriers to donate kidneys from related donors were mentioned differently. One-hundred and eight of 152 potential donors (71%) were not inclined to donate because kidneys could be bought from non-related donor; 53 (35%) had fear of not being able to maintain their family and raise their children; 29 (26%) had fear of having post-operative renal and non-renal complications; 86 (57%) gave more than one reason and another 25 (17%) have objected to donate for non-declared reasons.

Since the recent government proclamation of January 2008, the number of public kidney transplant centers has increased nationwide from four in 2007 to seven by the end of 2008 after the establishment of new public transplant centers in three largest cities of Syria which are Al-Assad University Hospital in Damascus, Al-Watani Hospital in Homs, and Ibn-Rochd Hospital in Aleppo. All these three centers together have performed 65 kidney transplants in 2008.

## DISCUSSION

The estimated incidence and prevalence of ESRD in Syria are 100 per year, and 200 pmp, respectively. Out of a Syrian population of 20 millions, the estimated number of new ESRD cases is about 2000 every year. The estimated prevalence of ESRD patients undergoing dialysis is 143 pmp [8]; thus, nearly 70% of all ESRD patients can benefit dialysis facilities. The 3-year survival rate of Syrian dialysis population has been estimated to be up to 64% [9] which, together with the persisting low rate of kidney transplantation, has led to a severe shortage of kidney supplies. Although the supply increased to 7 pmp in 2002, it remained fairly insufficient and responded only to 10% of the demand which was estimated to be around 75 kidneys pmp per year [9]. Consequently, a substantial increase in the rate of kidney transplantation performed on Syrian nationals abroad was observed from 8% of all kidney transplantations in 1988 to 65% in 1998 when it reached its highest share. Subsequently, it diminished again to 20%–45% in the early 2000s (Table 1) even before the legislation of 2003 was enacted to lessen the organ shortage by using organs from unrelated donors.

As a result of the 2003 national Syrian legislation, the kidney transplant rate has increased from seven kidney transplants pmp in 2002 to more than 17 in 2007. In the meantime, the rate of kidney transplantation performed on Syrian nationals abroad has declined to less than 2% in 2007 (Table 1).

**Figure 2:**
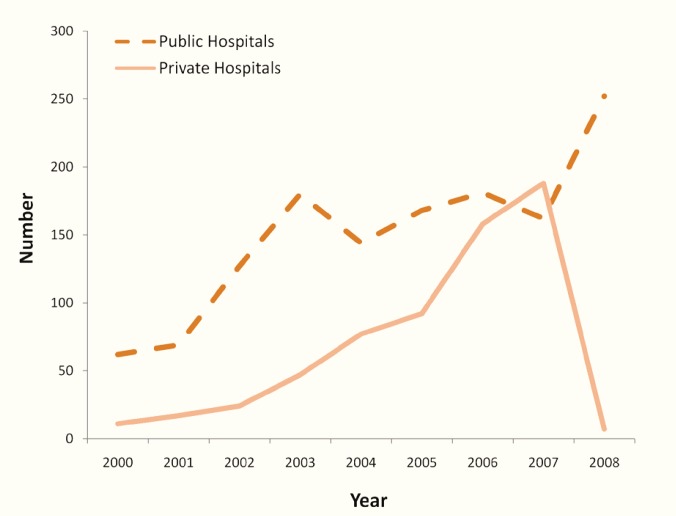
Evolution of kidney transplantation in public vs private hospitals

The practice of unrelated kidney donation by living volunteers has been mostly flourishing in private hospitals where the vast majority of donors were unrelated to recipients and considered as the major contributor to the substantial increase in the kidney transplant rate of more than nine-fold noticed in private sector in five years (2002–2007) (Fig. 2). The growing practice of unrelated donation in private sector has been in fact at the expense of decreasing the percentage of related donors to as little as 8% of all donors in private sector as compared to 50% in public sector. Meanwhile, the kidney transplant rate in public sector did not increase following the 2003 legislation, but rather declined to 8 pmp in 2007 as compared to 9.5 pmp in the same year in private sector. This indicates a steady trend toward expanding kidney transplant activities from unrelated donors in private sector and decreasing the rate of kidney transplant in public sector where the majority of potentially related donors (71%) were not inclined to donate kidneys as long as kidneys could be bought from a non-related donor.

Reversing that trend was in fact one of the major concerns for health authorities because such a trend could lead to possible closure of several public kidney transplant centers in which kidney vendors are not welcomed if the legislation was not changed.

The practice of “kidney selling,” although prohibited, has quickly become a common and readily available source of organs, and vendors have found ways to sell their kidneys through disreputable brokers, especially in the private sector. This practice has raised ethical concerns regarding organ commercialism, exploitation of poor, and undermining public trust of the transplant system.

Since we are in a global struggle to combat organ commercialism and considering the principles expressed in the World Health Assembly (WHA) 57.18 resolution on human organ and tissue transplantation of May 2004 [10], which called upon member states to “take measures to protect the poorest and vulnerable groups from transplant tourism and the sale of tissues and organs.” In January 2008, following detailed discussion and exchange of thoughts and opinions on how to approach and offset this dilemma to take the necessary actions that would fulfill the direction of the aforementioned resolution, the government of Syria issued a pronouncement prohibiting kidney transplantation in private sector. Thereafter, only public hospitals were authorized to perform kidney transplantation.

In the meantime, the ministry of health has set up a new legal framework governing donation and transplantation activities in the public sector in a way to make the donated organs as community resources and not to be marketed for financial gain. Later, the ministry formed independent commissions including physicians, lawyers, and psychiatrists with a main task of interviewing both recipients and donors to see whether the essential conditions are met.

Since this 2008, the administrative order was promulgated, and with the establishment of new public transplant centers in the three largest cities in Syria, the kidney transplant rate in public hospitals has substantially increased by 55%. Consequently, the rate of kidney transplants performed abroad decreased from eight in 2007 to five in 2008. Syria should be considered as an important model in the region, and with the background of the Istanbul declaration [11] and the revised Guiding Principles of the WHO, the national Syrian pronouncement of 2008 is considered to be an important testimony.

## CONCLUSION

The pronouncement by Syria becomes an important testimony and resolves to fulfill the WHA resolution of 2004, but still the recommendations of the Istanbul declaration have yet to be implemented in Syria. However, the expansion of kidney transplant centers in the public sector is an important initial step in initiating a deceased organ donation program that has to be established to lessen the burden of living donors and to enable a national self-sufficiency not only in kidney but in all other organs and tissues.
